# Uremia alters HDL composition and cholesterol efflux capacity

**DOI:** 10.1186/2050-6511-13-S1-A15

**Published:** 2012-09-17

**Authors:** Michael Holzer, Ruth Birner-Grünberger, Tatjana Stojaković, Dalia El-Gamal, Veronika Binder, Christian Wadsack, Ákos Heinemann, Gunther Marsche

**Affiliations:** 1 Institute of Experimental and Clinical Pharmacology, Medical University of Graz, 8010 Graz Austria; 2 Proteomics Core Facility, Centre for Medical Research, Medical University of Graz, 8036 Graz, Austria; 3 Clinical Institute of Medical and Chemical Laboratory Diagnostics, Medical University of Graz, 8036 Graz, Austria; 4 Department of Obstetrics and Gynecology, Medical University of Graz, 8036 Graz, Austria

## Background

Functional impairment of HDL may contribute to the excess cardiovascular mortality experienced by patients with renal disease, but the effect of advanced renal disease on the composition and function of HDL is not well understood.

## Methods

Mass spectrometry and biochemical analyses were used to study alterations in the proteome and lipid composition of HDL isolated from patients on maintenance hemodialysis.

## Results

We identified a significant increase in the amount of acute-phase protein serum amyloid A1, albumin, lipoprotein-associated phospholipase A_2_, and apoC-III composing uremic HDL. Furthermore, uremic HDL contained reduced phospholipids and increased triglycerides and lysophospholipids. With regard to function, these changes impaired the ability of uremic HDL to promote cholesterol efflux from macrophages.

## Conclusions

In summary, the altered composition of HDL in renal disease seems to inhibit the cardioprotective properties of HDL. Assessing HDL composition and function in renal disease may help to identify patients at increased risk for cardiovascular disease.

